# Diabetes risk and amino acid profiles: cross-sectional and prospective analyses of ethnicity, amino acids and diabetes in a South Asian and European cohort from the SABRE (Southall And Brent REvisited) Study

**DOI:** 10.1007/s00125-015-3517-8

**Published:** 2015-02-19

**Authors:** Therese Tillin, Alun D. Hughes, Qin Wang, Peter Würtz, Mika Ala-Korpela, Naveed Sattar, Nita G. Forouhi, Ian F. Godsland, Sophie V. Eastwood, Paul M. McKeigue, Nish Chaturvedi

**Affiliations:** 1 UCL Institute of Cardiovascular Science, 170 Tottenham Court Road, London, W1T 7HA UK; 2 Computational Medicine, Institute of Health Sciences, University of Oulu, Oulu, Finland; 3 NMR Metabolomics Laboratory, School of Pharmacy, University of Eastern Finland, Kuopio, Finland; 4 Oulu University Hospital, Oulu, Finland; 5 Computational Medicine, School of Social and Community Medicine and the Medical Research Council Integrative Epidemiology Unit, University of Bristol, Bristol, UK; 6 Institute of Cardiovascular and Medical Sciences, University of Glasgow School of Medicine, Glasgow, UK; 7 MRC Epidemiology Unit, University of Cambridge School of Clinical Medicine, Institute of Metabolic Science, Cambridge, UK; 8 Endocrinology and Medicine, Department of Medicine, Imperial College London, London, UK; 9 Centre for Population Health Sciences, University of Edinburgh, Edinburgh, UK

**Keywords:** Amino acids, Cohort, Diabetes, Ethnicity, European, South Asian

## Abstract

**Aims/hypothesis:**

South Asian individuals have an increased risk of diabetes compared with Europeans that is unexplained by obesity and traditional or established metabolic measures. Circulating amino acids (AAs) may provide additional explanatory insights. In a unique cohort of European and South Asian men, we compared cross-sectional associations between AAs, metabolic and obesity traits, and longitudinal associations with incident diabetes.

**Methods:**

Nuclear magnetic spectroscopy was used to measure the baseline (1988–1991) levels of nine AAs in serum samples from a British population-based cohort of 1,279 European and 1,007 South Asian non-diabetic men aged 40–69 years. Follow-up was complete for 19 years in 801 European and 643 South Asian participants.

**Results:**

The serum concentrations of isoleucine, phenylalanine, tyrosine and alanine were significantly higher in South Asian men, while cross-sectional correlations of AAs with glycaemia and insulin resistance were similar in the two ethnic groups. However, most AAs were less strongly correlated with measures of obesity in the South Asian participants. Diabetes developed in 227 (35%) South Asian and 113 (14%) European men. Stronger adverse associations were observed between branched chain and aromatic AAs and incident diabetes in South Asian men. Tyrosine was a particularly strong predictor of incident diabetes in South Asian individuals, even after adjustment for metabolic risk factors, including obesity and insulin resistance (adjusted OR for a 1 SD increment, 1.47, 95% CI 1.17,1.85, *p* = 0.001) compared with Europeans (OR 1.10, 0.87, 1.39, *p* = 0.4; *p* = 0.045 for ethnicity × tyrosine interaction).

**Conclusions/interpretation:**

Branched chain and aromatic AAs, particularly tyrosine, may be a focus for identifying novel aetiological mechanisms and potential treatment targets for diabetes in South Asian populations and may contribute to their excess risk of diabetes.

**Electronic supplementary material:**

The online version of this article (doi:10.1007/s00125-015-3517-8) contains peer-reviewed but unedited supplementary material, which is available to authorised users.

## Introduction

The global burden of type 2 diabetes is set to rise exponentially, with the Indian subcontinent predicted to contribute the greatest increase in the number of people with diabetes by 2030 [1]. South Asian migrant populations also experience a greater burden of diabetes than their host populations of white European origin [2, 3]. The causal mechanisms underlying progression to type 2 diabetes remain poorly understood, and no study has yet compellingly explained the reasons for the excess risk of diabetes experienced by South Asian individuals, suggesting that complex metabolic disturbances may underlie the ethnic difference [3].

Many recent studies using metabolite profiling in European-origin populations have suggested five branched chain and aromatic amino acids (AAs) as predictors of insulin resistance and the future onset of type 2 diabetes [4–9]. A combination of three AAs predicted future diabetes in the Framingham Offspring Study, and this finding was replicated in an independent prospective cohort and in a random cohort sample [5]. More recently, a study of over 9,000 Finnish men revealed that increasing glycaemia was associated with increasing levels of six AAs (alanine, isoleucine, leucine, valine, phenylalanine and tyrosine) and with decreasing levels of histidine and glutamine; these AA associations were almost fully explained by insulin sensitivity [10].

Associations of AA profiles with insulin resistance were reported in a small cross-sectional study of 263 South Asian and Chinese men living in Singapore. The findings suggested that perturbations in AA homeostasis and increased protein turnover might underlie insulin resistance in these Chinese and South Asian men [7]. To our knowledge, there is only one other published study of AA profiles in association with insulin resistance or diabetes in South Asian individuals [11], and no studies that provide longitudinal data.

In a population-based cohort of non-diabetic British European and South Asian men followed up for 19 years, we compared by ethnicity nine circulating AAs (isoleucine, leucine, valine, phenylalanine, tyrosine, alanine, glutamine, glycine and histidine) in association with markers of insulin resistance and obesity, both in a cross-sectional analysis and as predictors of incident diabetes. Given the paucity of such studies in non-European populations, we also attempted a replication in South Asian men of the widely cited Framingham Offspring Study [5] with regard to AAs in association with incident diabetes.

## Methods

The SABRE (Southall And Brent REvisited) Study involves a community-based cohort of European, South Asian and African-Caribbean origin living in North and West London. Details of the cohort have been published elsewhere [12]. Participants aged 40–69 years at baseline (1988–1991) were randomly selected from age- and sex-stratified primary care physician lists (*n* = 4,063) and workplaces (*n* = 795) in the London districts of Southall and Brent. As primary care registration is free and provides access to all health services in the UK, this forms a representative and comprehensive sampling frame. The baseline study was designed to investigate cardiometabolic risk in different ethnic groups, primarily in men. The current analyses are restricted to South Asian and European men recruited to the Southall arm of the study (1988–1990), due to the availability of stored baseline serum samples. We excluded those with diabetes at the baseline visit.

All the South Asian participants were migrants originating from the Indian subcontinent (52% Punjabi Sikh, 20% Hindu, 15% Muslim and 13% other South Asian). Interviewers recorded ethnicity on the basis of physical appearance, country of birth, name and parental origins supplemented with direct enquiry in cases of doubt. At baseline (1988–1990), the participants underwent fasting (morning) blood tests, BP measurements and anthropometry, and completed a health and lifestyle questionnaire. The questionnaire included the frequency of alcohol consumption and food frequency covering the major food groups consumed over the previous week. Physical activity was assessed by questionnaire, giving a summary estimate of weekly energy expenditure in daily activities plus sport, walking, cycling and strenuous activities [13]. An OGTT was also performed at the baseline visit [12]. Serum samples from participants attending the Southall study centres were stored at −80°C.

Deaths were flagged by the Office for National Statistics. During the period 2008–2011, survivors were invited to participate in a follow-up, including health and lifestyle questionnaires, a review of the primary care medical record and/or clinic attendance at St Mary’s Hospital, London. Clinic attendees fasted overnight and underwent measurements as at baseline.

All the participants gave their written informed consent. Approval for the baseline study was obtained from Ealing, Hounslow and Spelthorne, Parkside and University College London research ethics committees, and at follow-up from the St Mary’s Hospital Research Ethics Committee (ref. 07/H0712/109).

### Identifying baseline and incident diabetes

The physician’s diagnosis or 1999 WHO criteria [14] for fasting and OGTT blood glucose measurements defined baseline diabetes as an exclusion criterion. Incident diabetes was identified from a positive report from at least one of the following sources:

1. A review of the primary care medical record that reported a diagnosis of diabetes or the prescription of any glucose-lowering medications
2. The participant questionnaire, with a recall of physician-diagnosed diabetes together with the year of diagnosis and/or the receipt of named glucose-lowering medication
3. Clinical follow-up at 20 years with fasting or OGTT plasma glucose results meeting the 1999 WHO criteria [14]; plasma glucose was measured using hexokinase/NADP methods (Abbott Diagnostics, Maidenhead, UK)
4. A death certificate for the participant.

For the OGTT, plasma glucose and insulin were measured both at baseline and at follow-up during fasting and at 2 h after the oral ingestion of 75 g glucose [12]. Hepatic insulin resistance (HOMA-IR) was estimated using the HOMA2 calculator [15], and the Matsuda index of insulin resistance (Matsuda-IR = inverse of insulin sensitivity calculated according to the methods of Matsuda and DeFronzo [16, 17]) provided an estimate of whole-body insulin resistance, based on the glucose and insulin values during fasting and at 2 h after the OGTT [16, 17].

### Other baseline measurements

Seated BP was recorded as the mean of two resting measurements. The participant’s height was measured using a stadiometer. The waist and hip circumferences were measured using a fibreglass tape with a spring balance set to a constant tension of 600 g. Harpenden calipers were used to a standard protocol to measure the skinfold thickness. Subcutaneous truncal fat was estimated by adding together the subscapular and suprailiac skinfold thicknesses.

### AA quantification

A high-throughput serum nuclear magnetic resonance (NMR) platform was used for AA quantification. The baseline fasting serum samples were stored at −80°C and thawed overnight in a refrigerator. A proton NMR spectrum was acquired in which spectral signals from macromolecules and lipoprotein lipids were suppressed to enhance the detection of AAs. The levels of nine AAs were quantified (isoleucine, leucine, valine, phenylalanine, tyrosine, alanine, glutamine, glycine and histidine) in mmol/L. The AA profiling has previously been used in large epidemiological studies [10], and the methodology has been described in detail elsewhere [18–20].

### Statistical analyses

We tabulated baseline conventional risk factors and the nine measured AAs in non-diabetic participants for cross-sectional analysis and in those for whom diabetes follow-up data were available. We show medians (interquartile ranges) because of the non-normal nature of many of the distributions. Variables with a non-normal distribution were natural log-transformed and standardised to 1 SD before further analysis. The results are presented for individual AAs, for the three-AA combination (isoleucine, phenylalanine and tyrosine) and for the five-AA combination (isoleucine, leucine, valine, phenylalanine and tyrosine) [5]. Summary variables were derived for combinations of AAs according to the formula: *z*-score of log *X*_1_ + *z*-score of log *X*_2_ + *z*-score of log *X*_3_, with *X*_*j*_ denoting the value of the *j*th AA. All the scores were thereafter scaled to 1 SD.

We examined the correlations between AAs, markers of obesity and insulin resistance, and glycaemia at baseline using non-parametric methods (Spearman’s rho). We tested ethnic differences in the correlations between AAs and markers of insulin resistance and obesity using Fisher’s *r* to *z* transformation [21].

Prospective analyses used logistic regression to describe age-adjusted associations between the AAs as continuous variables and incident diabetes using ethnicity-specific models. The use of logistic regression for the primary analyses avoided the loss of cases with missing dates of diagnosis and improved the comparison with findings of the Framingham Offspring Study [5]. The associations between AAs and incident diabetes were further tested using quartiles of AA concentrations. The analyses were adjusted for age together with a group of prespecified conventional risk factors that had been identified in our earlier published analyses as important mediators of ethnic differences in the incidence of diabetes (WHR, truncal skinfold thickness, Matsuda-IR, smoking and HDL-cholesterol level) [3]. We further added the self-reported number of units of alcohol consumption to this model as alcohol consumption has been shown to affect AA levels [22]. We assessed the interactions between ethnicity and each AA in association with incident diabetes, and examined the effects of adjustment for AAs and prespecified covariates on the ethnic differences in risk of diabetes. For comparability with the Framingham Offspring Study, model 5 was adjusted for age, fasting blood glucose and BMI.

We compared the predictive powers of AAs and conventional risk factors for risk of diabetes using the net reclassification index (NRI), the integrated improvement index (IDI) and Harrell’s *C* statistic [23, 24]. NRI cut-off points of 15%/30% were selected a priori to reflect the high incidence rates observed as there are no thresholds for intervention with regard to diabetes risk.

In the sensitivity analyses, we repeated the above analyses of associations with incident diabetes as follows:

1. Further adjustment of model 4 for the frequency of meat/fish, dairy and green vegetable consumption and physical activity, and for serum creatinine as a marker of renal function
2. Separate analyses for the three largest South Asian subgroups (the Sikh [52%], Hindu [20%] and Muslim [15%] groups)
3. The use of competing risks regression (where the competing risk was death from other causes) based on Fine and Gray’s proportional subhazards methods [25].

Statistical significance was defined as *p* < 0.05. We did not apply a correction for multiple testing as the associations between the AAs and glycaemic risk factors and risk of diabetes are established in populations of European origin [5, 8, 10, 26]. Statistical analyses were conducted using Stata v13 statistical software (StataCorp, College Station, TX, USA).

## Results

**Fig. 1:**
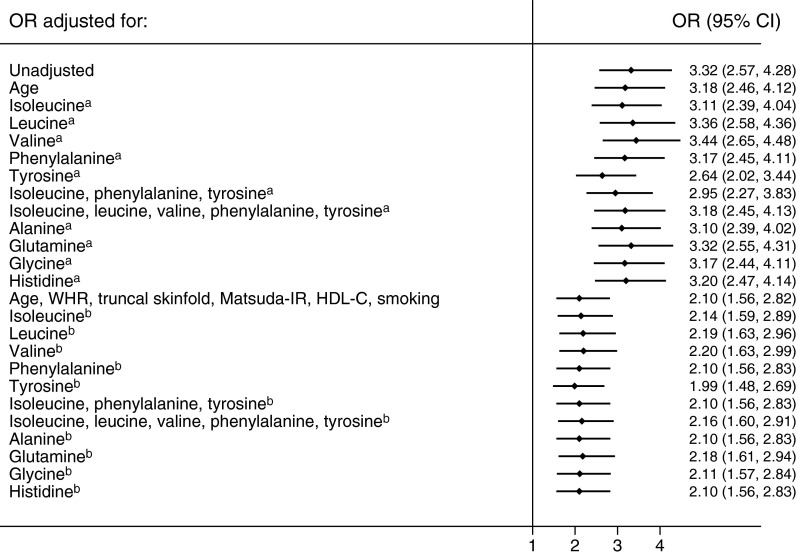
ORs for ethnic differences in the incidence of diabetes in South Asian vs European men, adjusted for age and AAs, and for conventional risk factors and AAs. ^a^Adjusted for age plus each AA or combination in turn. ^b^Adjusted for age, WHR, truncal skinfold thickness, Matsuda-IR, HDL-cholesterol (HDL-C) level and current smoking, plus each AA or combination in turn

Of 1,515 European and 1,419 South Asian male study participants from the Southall study centres, 1,423 (94%) and 1,111 (78%), respectively, did not have diabetes at baseline and were included in this study. Serum samples were available for 1,279 European men and 1,007 South Asian men, of whom 801 (63%) Europeans and 643 (64%) South Asians had known follow-up information for diabetes status (electronic supplementary material [ESM] Fig. 1).

### Baseline cross-sectional analyses (Southall centre: 1988–1990)

South Asian men were more insulin resistant and more had diagnosed hypertension compared with European men. They were more centrally obese but had lower a BMI. They were less physically active, consumed less alcohol and smoked less. They also reported a lower consumption of meat, fish and dairy products (Table 1). The serum concentrations of isoleucine, phenylalanine, tyrosine and alanine were significantly higher in South Asian men (Table 2). The findings were almost identical in those with and without information on diabetes status at follow-up. No participants were receiving lipid-lowering medications at baseline. Among the South Asian men, the concentrations of branched chain AAs and histidine were higher in Muslims than in Sikhs or Hindus, while the glutamine and glycine concentrations were higher in the Hindu than the Sikh or Muslim participants; however, the ethnic differences in AA levels compared with Europeans were maintained regardless of the South Asian subgroup.

**Table 1:** Baseline characteristics of European and South Asian men without baseline diabetes

Baseline characteristics	All with stored baseline serum (included in cross-sectional analyses)			All with stored baseline serum and data regarding diabetes status at follow-up (included in prospective analyses)		
	European	South Asian	*p* values for ethnic difference	European	South Asian	*p* values for ethnic difference
Number of individuals	1,279	1,007		801	643	
Age, mean ± SD	52.9 ± 7.3	50.6 ± 7.0	<0.001	52.6 ± 7.2	50.6 ± 7.0	<0.001
Fasting glucose (mmol/l)	5.36 (5.04, 5.69)	5.46 (5.09, 5.83)	<0.001	5.36 (5.03, 5.71)	5.45 (5.06, 5.82)	<0.001
2 h glucose (mmol/l)	4.85 (4.04, 5.66)	5.34 (4.50, 6.20)	<0.001	4.86 (4.04, 5.72)	5.27 (4.45, 6.08)	<0.001
Fasting insulin (pmol/l)	50.00 (33.34, 72.23)	69.80 (47.92, 98.62)	<0.001	51.39 (34.03, 73.62)	69.10 (47.92, 98.62)	<0.001
2 h insulin (pmol/l)	134.04 (79.87, 234.05)	274.67 (158.35, 504.90)	<0.001	141.68 (33.34, 235.44)	275.37 (159.74, 497.26)	<0.001
HDL-cholesterol (mmol/l)	*n* = 1,272 1.24 (1.05, 1.48)	*n* = 1,000 1.15 (0.98, 1.35)	<0.001	*n* = 795 1.24 (1.06, 1.48)	*n* = 640 1.15 (0.99, 1.37)	<0.001
Triacylglycerols (mmol/l)	1.43 (1.02, 2.09)	1.71 (1.15, 2.51)	<0.001	1.36 (0.99, 2.0)	1.60 (1.09, 2.31)	<0.001
Serum creatinine (μmol/l)	60.37 (54.60, 67.24)	57.47 (51.43, 64.03)	<0.001	60.79 (55.0, 67.79)	57.47 (51.78, 63.15)	<0.001
HOMA-IR	0.8 (0.5, 1.2)	1.1 (0.8, 1.6)	<0.001	0.8 (0.6, 1.2)	1.1 (0.8, 1.6)	<0.001
Matsuda-IR	0.20 (0.13, 0.32)	0.35 (0.22, 0.58)	<0.001	0.21 (0.13, 0.33)	0.35 (0.22, 0.57)	<0.001
Waist circumference (cm)	90.9 (84.5, 97.7)	92.1 (86.1, 98.5)	0.02	90.6 (84.7, 97.5)	91.5 (85.7, 97.6)	0.06
WHR	0.94 (0.90, 0.98)	0.98 (0.93, 1.02)	<0.001	0.94 (0.90, 0.98)	0.98 (0.93, 1.02)	<0.001
Truncal skinfold thickness (cm)	3.7 (2.9, 4.7)	4.5 (3.7, 5.5)	<0.001	3.7 (2.9, 4.7)	4.5 (3.7, 5.7)	<0.001
BMI (kg/m^2^)	25.65 (23.68, 27.96)	25.46 (23.50, 27.53)	0.03	25.63 (23.69, 27.78)	25.28 (23.30, 27.23)	0.05
Systolic BP (mmHg)	121 (111, 132)	122 (112, 133)	0.04	119 (110, 132)	121 (112, 133)	0.02
Treated hypertension	103 (8)	117 (12)	0.004	63 (8)	81 (13)	0.003
Smoking, never/ex/current (%)	26/41/33	74/9/16	<0.001	28/40/32	74/10/16	<0.001
Alcohol (units per week)	*n* = 1,244 11 (3, 23)	*n* = 969 3 (0, 15)	<0.001	*n* = 774 12 (3, 24)	*n* = 619 3 (0, 14)	<0.001
Meat and fish consumption during previous 7 days, quartiles of frequency, lowest to highest (%)	4/15/40/41	35/26/25/13	<0.0001	3/14/40/42	36/26/24/15	<0.0001
Dairy product consumption during previous 7 days, none/1 day/2–3 days/most days (%)	2/4/11/83	7/8/19/66	<0.0001	2/4/11/83	6/6/20/67	<0.0001
Green vegetable consumption during previous 7 days, none/1 day/2–3 days/most days (%)	7/7/32/54	4/8/35/53	0.017	6/7/31/56	4/8/36/51	0.057
Physical activity (kJ × 10^3^/week)	11 (7, 16)	9 (5, 13)	<0.001	11 (7, 16)	9 (5, 13)	<0.001
Education (years)	10 (9, 11)	12 (10, 15)	<0.001	10 (9, 11)	12 (10, 15)	<0.001

Data are median (interquartile range), *n* (%) unless otherwise stated

**Table 2:** Baseline AA levels of European and South Asian men (all without baseline diabetes)

Amino acids (mmol/l)	All with stored baseline serum (included in cross-sectional analyses)			All with stored baseline serum and data regarding diabetes status at follow-up (included in prospective analyses)		
Baseline characteristics	European	South Asian	*p* values for ethnic difference	European	South Asian	*p* values for ethnic difference
Number	1,279	1,007		801	643	
Isoleucine	0.057 (0.050, 0.066)	0.060 (0.052, 0.068)	<0.0001	0.058 (0.050, 0.066)	0.060 (0.052, 0.068)	0.002
Leucine	0.091 (0.080, 0.11)	0.093 (0.083, 0.11)	0.17	0.092 (0.081, 0.11)	0.092 (0.083, 0.10)	0.8
Valine	0.179 (0.158, 0.202)	0.178 (0.156, 0.199)	0.17	0.180 (0.158, 0.204)	0.176 (0.155, 0.198)	0.017
Phenylalanine	0.092 (0.084, 0.10)	0.094 (0.086, 0.10)	0.019	0.092 (0.084, 0.10)	0.094 (0.085, 0.10)	0.12
Tyrosine	0.054 (0.048, 0.061)	0.060 (0.053, 0.067)	<0.0001	0.054 (0.048, 0.061)	0.059 (0.052, 0.067)	<0.0001
Isoleucine, phenylalanine, tyrosine combined	0.205 (0.186, 0.2228)	0.213 (0.197, 0.234)	<0.0001	0.205 (0.186, 0.228)	0.213 (0.197, 0.233)	<0.0001
Alanine	0.326 (0.290, 0.365)	0.335 (0.301, 0.375)	<0.0001	0.328 (0.290, 0.367)	0.335 (0.302, 0.374)	0.001
Glutamine	0.384 (0.257, 0.448)	0.410 (0.309, 0.471)	<0.0001	0.385 (0.262, 0.450)	0.415 (0.317, 0.473)	<0.0001
Glycine	*n* = 1,267 0.288 (0.262, 0.318)	*n* = 998 0.282 (0.256, 0.311)	0.006	*n* = 794 0.287 (0.262, 0.316)	*n* = 638 0.282 (0.257, 0.312)	0.10
Histidine	0.077 (0.069, 0.088)	0.078 (0.070, 0.089)	0.3	0.077 (0.068, 0.088)	0.078 (0.070, 0.090)	0.14

Data are median (interquartile range)

Positive and significant correlations with markers of glycaemia and insulin resistance were observed for isoleucine, leucine, valine, phenylalanine, tyrosine and alanine in both ethnic groups. The correlations were non-significantly weaker between AAs and glycaemia and insulin resistance in South Asian men. Histidine was weakly positively correlated with measures of glycaemia and insulin resistance, and glycine and glutamine were negatively correlated in both ethnic groups. Most AAs were less strongly correlated with obesity measures in South Asian men (significantly so for branched chain AAs and alanine) (Table 3).

**Table 3:** Correlations between AAs and markers of glycaemia/insulin resistance and obesity at baseline in non-diabetic men

Spearman’s *ρ* ^a^	European men (*n* = 1,279) South Asian men (*n* = 1,007)	Fasting glucose	2 h glucose	Fasting insulin	2 h insulin	HOMA-IR	Matsuda-IR	WHR	Waist circumference	BMI	Truncal skinfold thickness
Isoleucine	European men	0.16	0.12	0.35	0.28	0.35	0.34	0.30	0.34	0.31	0.32
	South Asian men	0.13	0.13	0.34	0.32	0.34	0.36	0.21^b^	0.25^b^	0.23^b^	0.30
Leucine	European men	0.18	0.10	0.30	0.20	0.31	0.27	0.23	0.27	0.27	0.29
	South Asian men	0.14	0.07	0.26	0.21	0.26	0.25	0.14^b^	0.19^b^	0.18^b^	0.25
Valine	European men	0.18	0.14	0.32	0.23	0.33	0.30	0.23	0.28	0.29	0.31
	South Asian men	0.15	0.12	0.26	0.27	0.27	0.29	0.13^b^	0.19^b^	0.22	0.29
Phenylalanine	European men	0.19	0.13	0.24	0.18	0.25	0.23	0.26	0.25	0.22	0.21
	South Asian men	0.09	0.11	0.21	0.18	0.21	0.22	0.22	0.22	0.15	0.20
Tyrosine	European men	0.22	0.11	0.36	0.26	0.36	0.33	0.32	0.35	0.34	0.31
	South Asian men	0.23	0.14	0.29	0.24	0.30	0.30	0.30	0.35	0.30	0.27
Alanine	European men	0.27	0.12	0.34	0.23	0.35	0.31	0.20	0.24	0.23	0.22
	South Asian men	0.24	0.15	0.27	0.20	0.29	0.27	0.17	0.16^b^	0.17	0.23
Glutamine	European men	−0.11	−0.09	−0.18	−0.07	−0.18	−0.14	−0.11	−0.12	−0.12	−0.16
	South Asian men	−0.07	−0.03^a^	−0.17	−0.04^a^	−0.17	−0.10	−0.08	−0.08	−0.07	−0.15
Glycine	European men (*n* = 1,267)	0.04^a^	−0.0^a^	−0.07	−0.10	−0.05	−0.09	−0.09	−0.10	−0.11	−0.05
	South Asian men (*n* = 998)	0.03^a^	0.003^a^	−0.06^a^	−0.09	−0.06^a^	−0.08	−0.08	−0.11	−0.15	−0.01^a^
Histidine	European men	0.08	0.08	0.11	0.04^a^	0.10	0.09	0.03^a^	0.07	0.07	0.14
	South Asian men	0.07^a^	0.03^a^	0.04^a^	0.01^a^	0.04^a^	0.03^a^	0.01^a^	0.02^a^	0.02^a^	0.16

^a^ *p* values for correlations within ethnic group were <0.05, unless marked ^a^

^b^ *p* values for ethnic group difference in correlation coefficient <0.05

### Prospective analyses: AAs and incident diabetes (1988–2011)

The median duration of follow-up was 19 years. Diabetes developed in 227 South Asian men (35%) and 113 European men (14%). The median (interquartile range) number of years from baseline to the development of diabetes was 15 (11, 18) in Europeans and 14 (9, 18) in South Asians (*p* = 0.075), and the median (interquartile range) age at diagnosis of diabetes was 67 (60, 71) years in the European participants and 62 (57, 68) years in the South Asian participants (*p* < 0.0002).

In logistic regression analyses adjusted only for age, all AAs and AA combinations were associated with incident diabetes in both ethnic groups (Table 4, model 1), with the exception of phenylalanine, glutamine and histidine in European men and glycine in South Asian men. Similar results were obtained when the AAs were analysed in quartiles (see ESM Table 1). Multivariable analyses for the European men showed that, with the exception of glycine and isoleucine, all linear associations (per SD log-transformed AA level) were markedly attenuated following adjustments for obesity and further attenuated on adjustment for insulin resistance and other conventional risk factors (Table 4, models 2–5). Positive associations between incident diabetes and individual AAs and the three- and five-AA combinations were generally more marked in South Asian individuals and only partially attenuated on adjustment for obesity measures and fasting glucose levels (Table 4, models 2 and 5).

**Table 4:** Associations between AAs and incident diabetes

OR per SD log-transformed AA concentration		European men	*p* values	South Asian men	*p* values	*p* values for interaction ethnicity × AA
Isoleucine	Model 1	1.48 (1.20, 1.83)	3 × 10^−4^	1.58 (1.30, 1.93)	5 × 10^−6^	0.7
	Model 2	1.27 (1.02, 1.58)	0.032	1.34 (1.10, 1.65)	0.004	0.6
	Model 3	1.09 (0.94, 1.00)	0.5	1.22 (0.98, 1.51)	0.069	0.7
	Model 4	1.09 (0.87, 1.38)	0.4	1.19 (0.95, 1.48)	0.13	0.6
	Model 5	1.21 (0.97, 1.51)	0.089	1.42 (1.16, 1.75)	0.001	0.3
Leucine	Model 1	1.30 (1.10, 1.55)	0.003	1.50 (1.23, 1.82)	6 × 10^−5^	0.3
	Model 2	1.19 (0.99, 1.43)	0.071	1.34 (1.10, 1.65)	0.004	0.4
	Model 3	1.09 (0.88, 1.34)	0.4	1.31 (1.06, 1.62)	0.014	0.2
	Model 4	1.10 (0.89, 1.35)	0.4	1.22 (0.98, 1.52)	0.074	0.3
	Model 5	1.11 (0.92, 1.35)	0.3	1.36 (1.11, 1.68)	0.004	0.17
Valine	Model 1	1.29 (1.06, 1.58)	0.013	1.53 (1.26, 1.84)	1 × 10^−5^	0.2
	Model 2	1.12 (0.90, 1.38)	0.3	1.35 (1.11, 1.64)	0.003	0.2
	Model 3	1.02 (0.81, 1.28)	0.9	1.24 (1.01, 1.54)	0.044	0.3
	Model 4	1.00 (0.80, 1.27)	0.9	1.24 (1.00, 1.54)	0.052	0.3
	Model 5	1.04 (0.84, 1.29)	0.7	1.32 (1.08, 1.62)	0.007	0.14
Phenylalanine	Model 1	1.13 (0.93, 1.37)	0.2	1.36 (1.12, 1.64)	0.002	0.19
	Model 2	1.00 (0.82, 1.22)	1.0	1.19 (0.98, 1.45)	0.076	0.2
	Model 3	0.91 (0.74, 1.11)	0.4	1.12 (0.91, 1.37)	0.3	0.2
	Model 4	0.91 (0.74, 1.12)	0.4	1.08 (0.88, 1.33)	0.5	0.2
	Model 5	0.97 (0.80, 1.18)	0.8	1.29 (1.06, 1.57)	0.011	0.054
Tyrosine	Model 1	1.44 (1.17, 1.76)	5 × 10^−4^	1.92 (1.57, 2.35)	3 × 10^−10^	0.049
	Model 2	1.22 (0.98, 1.51)	0.071	1.66 (1.33, 2.03)	5 × 10^−6^	0.053
	Model 3	1.07 (0.85, 1.35)	0.6	1.55 (1.24, 1.93)	1 × 10^−4^	0.038
	Model 4	1.10 (0.87, 1.39)	0.4	1.47 (1.17, 1.85)	0.001	0.045
	Model 5	1.11 (0.90, 1.39)	0.3	1.56 (1.26, 1.93)	5 × 10^−5^	0.047
Alanine	Model 1	1.27 (1.03, 1.55)	0.022	1.45 (1.20, 1.75)	1 × 10^−4^	0.3
	Model 2	1.13 (0.91, 1.39)	0.3	1.31 (1.08, 1.59)	0.005	0.3
	Model 3	1.06 (0.85, 1.33)	0.6	1.21 (0.99, 1.49)	0.057	0.5
	Model 4	1.12 (0.89, 1.40)	0.3	1.15 (0.94, 1.42)	0.17	0.8
	Model 5	1.03 (0.84, 1.28)	0.8	1.25 (1.03, 1.51)	0.025	0.2
Glutamine	Model 1	0.91 (0.76, 1.09)	0.3	0.82 (0.70, 0.97)	0.022	0.4
	Model 2	0.96 (0.79, 1.16)	0.6	0.86 (0.73, 1.03)	0.10	0.4
	Model 3	0.98 (0.80, 1.19)	0.8	0.85 (0.71, 1.03)	0.091	0.2
	Model 4	0.94 (0.77, 1.15)	0.5	0.94 (0.77, 1.14)	0.5	0.4
	Model 5	0.94 (0.77, 1.15)	0.6	0.88 (0.73, 1.05)	0.15	0.6
Glycine	Model 1	0.77 (0.63, 0.93)	0.007	0.95 (0.80, 1.12)	0.5	0.10
	Model 2	0.78 (0.64, 0.96)	0.016	0.98 (0.82, 1.17)	0.8	0.11
	Model 3	0.75 (0.61, 0.93)	0.010	0.99 (0.82, 1.20)	0.9	0.047
	Model 4	0.76 (0.61, 0.94)	0.011	0.98 (0.81, 1.19)	0.8	0.058
	Model 5	0.78 (0.64, 0.96)	0.019	0.96 (0.80, 1.15)	0.7	0.13
Histidine	Model 1	0.98 (0.82, 1.18)	0.8	1.19 (0.99, 1.43)	0.062	0.15
	Model 2	0.94 (0.78, 1.15)	0.6	1.16 (0.96, 1.41)	0.13	0.15
	Model 3	0.93 (0.75, 1.15)	0.5	1.20 (0.98, 1.46)	0.084	0.077
	Model 4	0.93 (0.75, 1.15)	0.6	1.15 (0.93, 1.42)	0.2	0.10
	Model 5	0.92 (0.75, 1.12)	0.4	1.13 (0.93, 1.38)	0.2	0.14
Isoleucine, phenylalanine, tyrosine	Model 1	1.39 (1.14, 1.69)	0.001	1.85 (1.50, 2.29)	1 × 10^−8^	0.051
	Model 2	1.18 (0.95, 1.46)	0.13	1.54 (1.23, 1.92)	1 × 10^−4^	0.083
	Model 3	1.02 (0.81, 1.27)	0.9	1.39 (1.10, 1.75)	0.001	0.096
	Model 4	1.03 (0.82, 1.29)	0.8	1.32 (1.04, 1.68)	0.022	0.10
	Model 5	1.10 (0.89, 1.36)	0.4	1.57 (1.26, 1.96)	5 × 10^−5^	0.029
Branched chain + aromatic AAs	Model 1	1.36 (1.13, 1.65)	0.001	1.79 (1.45, 2.20)	5 × 10^−8^	0.061
	Model 2	1.18 (0.96, 1.45)	0.11	1.51 (1.21, 1.87)	1 × 10^−5^	0.10
	Model 3	1.04 (0.83, 1.29)	0.8	1.38 (1.10, 1.73)	0.006	0.12
	Model 4	1.05 (0.84, 1.30)	0.7	1.31 (1.04, 1.66)	0.023	0.13
	Model 5	1.10 (0.89, 1.35)	0.4	1.52 (1.22, 1.89)	2 × 10^−4^	0.040

Model 1: adjusted for age

Model 2: adjusted for age, WHR and truncal skinfold thickness

Model 3: adjusted for age, WHR, truncal skinfold thickness, Matsuda-IR, HDL-cholesterol level and current smoking

Model 4: model 3 plus alcohol consumption

Model 5: adjusted for age, fasting glucose level and BMI (for comparison with the Framingham Offspring Study [5])

Additional adjustment for Matsuda-IR, smoking and HDL-cholesterol level and then alcohol consumption resulted in further attenuation for branched chain AAs, phenylalanine, alanine, glutamine and histidine (models 3 and 4). However, the association for tyrosine was particularly marked among the South Asian participants even after full adjustment (fully adjusted OR for a 1 SD increment in the log-transformed level: 1.47 [1.17, 1.85] vs Europeans: 1.10 [0.87, 1.39]; ethnicity × tyrosine interaction *p* = 0.045 (fully adjusted)). Similarly, the three- and five-AA combinations of isoleucine, leucine, valine, phenylalanine and tyrosine were also more strongly associated with incident diabetes in South Asian participants (fully adjusted ORs [model 4]: South Asians: 1.32 [1.04, 1.68] and 1.31[1.04, 1.66] vs European participants: 1.03 [0.82, 1.29] and 1.05 [0.84, 1.30]), with weak evidence of ethnicity interactions (*p* = 0.10 and 0.13, respectively) (Table 4, model 4). Glycine was negatively associated with incident diabetes in European but not in South Asian men (interaction *p* = 0.06).

Comparisons with the Framingham Offspring Study are shown following adjustment for age, fasting glucose level and BMI (Table 4, model 5). In European men, the model 5 adjusted ORs per 1 SD increment in the log-transformed three-AA (isoleucine, phenylalanine and tyrosine) and five-AA (isoleucine, leucine, valine, phenylalanine and tyrosine) combinations were virtually identical and weak: 1.10 (0.89, 1.36) and 1.10 (0.89, 1.35). In contrast, the ORs were greater in South Asian men (1.57 [1.26, 1.96] and 1.52 [1.22, 1.89], respectively; Table 4, model 5). In South Asian men, the ORs for the upper vs lowest quartile were 3.11 (1.71, 5.67) and 2.55 (1.46, 4.48) for the three- and five-AA combinations, respectively, with evidence of linear associations. The corresponding categorical associations for Europeans were weaker (ORs for the upper vs lower quartile 1.99 [1.07, 3.67] and 1.98 [1.05, 3.76], respectively) (ESM Table 1, model 5).

In aiming to predict incident diabetes, no improvements in the C statistic, NRI or IDI were observed in European men on the addition of tyrosine or either the three- or five-AA combination to models containing prespecified conventional risk factors. However, in South Asian men, the IDI and NRI significantly improved with the addition of tyrosine, although only discrimination (IDI) significantly improved with the addition of the three- and five-AA combinations (ESM Table 2).

### Prospective analyses: AAs and ethnic differences in incidence of diabetes

In age-adjusted models, South Asian men had a 3.18-fold (95% CI 2.46, 4.12, *p* = 1.5 × 10^−18^) greater risk of developing diabetes than European men (Fig. 1). Additional adjustment for baseline tyrosine level reduced the ethnic difference in risk of diabetes to the greatest extent (OR 2.64 [2.02, 3.44]; 17% reduction). Adjustment for the prespecified combination of conventional risk factors reduced the ethnic OR to 2.10 (1.56, 2.82). Further adjustment for AAs modestly altered the ethnic difference in risk of diabetes; the addition of tyrosine to the full model led to the largest reduction in OR, to 1.99 (1.48, 2.69; 5% reduction) (Fig. 1).

### Sensitivity analyses

Sensitivity analyses that included adjustment for diet, physical activity or serum creatinine, or excluded participants with impaired fasting glucose or impaired glucose tolerance, produced similar results to those reported above. There were no significant age-adjusted interactions between the main South Asian subgroups (Punjabi Sikh, Hindu and Muslim) and AAs in association with incident diabetes (all interaction *p* values > 0.10).

Prospective analyses using competing risks regression resulted in the loss of 23 cases of incident diabetes without dates of diagnosis, but demonstrated similar ethnic differences in the incidence of diabetes (age-adjusted subhazard ratio [SHR] for South Asian vs European participants 2.90 [2.33, 3.61]), which were again most markedly attenuated on the addition of baseline tyrosine (SHR 2.52 [2.00, 3.20]). Competing risks regression also demonstrated similar associations between AAs and incident diabetes, for example the age-adjusted SHR per SD log-transformed tyrosine in Europeans of 1.31 (1.05, 1.64) and in South Asians of 1.65 (1.44, 1.89).

## Discussion

We report novel ethnicity-specific data regarding the associations between AAs and incident diabetes in over 19 years of follow-up. We demonstrate significant positive associations between baseline branched chain AAs, tyrosine and alanine and incident diabetes in middle-aged European men in accord with previous studies [4–9, 27]. In South Asian men, the same AAs, plus phenylalanine, were more strongly associated with incident diabetes compared with Europeans. In European men, obesity and insulin resistance, together with age, smoking, HDL-cholesterol level and alcohol consumption partially or completely accounted for the majority of associations between AAs incident diabetes. In contrast, in South Asian men, the adverse associations were, with the exception of phenylalanine, generally only moderately attenuated on adjustment for insulin resistance, smoking, HDL-cholesterol, alcohol intake and creatinine level, and remained statistically significant. Tyrosine and the combination of branched chain and aromatic AAs were particularly strongly associated with incident diabetes even after multivariable adjustment in South Asian men. Adjustment for tyrosine made a small (approximately 5%) contribution to explaining the ethnic difference in the incidence of diabetes.

Cross-sectional associations at baseline between AAs and markers of insulin resistance and glycaemia generally followed the patterns observed for incident diabetes, with the exception of histidine, which was minimally associated with markers of insulin resistance in South Asian men. Associations between branched chain AAs and obesity, although present, were weaker in South Asian than European participants. The latter findings, together with the weaker attenuation of AA associations with incident diabetes, suggest that conventional measures may not capture the best indicators of diabetes risk in South Asian individuals.

### Comparison with other studies

Although it did not have European comparators and was only a small cross-sectional analysis, the study by Tai et al [7] reported strong associations in South Asian and Chinese men of relatively low body weight between insulin resistance and branched chain and aromatic AAs and for a combination of isoleucine, leucine, phenylalanine, tyrosine and methionine. These associations were independent of dietary protein intake and BMI [7]. This is compatible with our cross-sectional finding of weaker associations between obesity and branched chain AAs in South Asian men, suggesting that increases in these AA levels may be driven by insulin resistance to a greater extent than obesity in South Asian individuals or that measures of obesity may be less satisfactory in this population.

Another small cross-sectional study in younger (age 35–45 years) Indian Asians living in India found strong correlations between isoleucine, leucine and phenylalanine and obesity in people with a high BMI both with and without type 2 diabetes, and lower correlations in those with a low BMI and no diabetes [11]. There was no comparator group, so we cannot compare the relative strength of the correlations. However, these findings are not inconsistent with ours, in that we also found significant correlations between branched chain and aromatic amino acids and measures of obesity, albeit weaker in the South Asian than in the European participants.

The findings of the Framingham Offspring Study for three AAs and for five AAs in the lower risk random cohort sample adjusted for age, fasting glucose level and BMI are comparable with our findings for European men for the upper vs lower quartiles of AA levels (although the linear association is attenuated), but they somewhat understate the associations observed in the higher risk South Asian men in our study [5]. We had anticipated that the findings in the European and South Asian participants in the SABRE Study would most closely resemble the Framingham random cohort. European SABRE participants had comparable baseline levels of glucose, insulin and obesity, but were younger, were less likely to be hypertensive and had lower triacylglycerol levels, perhaps explaining the somewhat weaker associations of AAs with incident diabetes compared with those observed in the Framingham random cohort. South Asian SABRE participants, who were also younger, were more centrally obese and had higher fasting glucose and insulin levels, but experienced similar triacylglycerol levels and less hypertension than the Framingham random cohort. Associations between AAs and incident diabetes in South Asian individuals more closely resembled the stronger associations seen in the Framingham replication cohort (Malmo Diet and Cancer cohort [28]) and Framingham high-risk propensity matched cases and controls, despite having markedly lower overall obesity (BMI) than the latter group [5].

### Possible mechanisms

Branched chain and aromatic AAs have been associated with insulin-resistant states, including diabetes, in many other studies in populations of mostly European origin [4, 5, 7, 9, 27]. Potential mechanisms include altered AA metabolism in the liver, kidneys, muscle or adipose tissue [29–31]. Serum creatinine as a marker of renal function did not explain associations between AAs and incident diabetes, nor did adjustment for alcohol intake, although we did not have other baseline measures of liver function. Two recent studies in European adults suggest that altered branched chain and aromatic AA metabolism is associated with impaired insulin sensitivity prior to the development of hyperglycaemia [26, 32]. South Asian individuals have lower muscle mass and more hepatic fat than Europeans [33–36]. Abdominal adipose tissue in South Asian individuals contains larger and more dysfunctional adipocytes [37]. It has also been suggested that increased AA levels may result from increased protein turnover in association with increased central obesity and reduced lean body mass [7]; this is a plausible, but untested, explanation for the increased levels of isoleucine, tyrosine, alanine and glutamine seen in our more centrally obese South Asian men.

It is not clear why associations between branched chain AAs and tyrosine and incident diabetes should be stronger in South Asian men. It is possible that this relates simply to the higher risk of baseline diabetes, but it is also possible that AA perturbations become more severe with a greater degree and/or longer duration of insulin resistance.

It is notable that tyrosine levels were substantially higher in the South Asian men, whereas ethnic differences in other AA levels were less marked. This suggests that higher tyrosine levels may not be due solely to increased insulin resistance in South Asian individuals.

There were no significant differences in the associations between AAs and incident diabetes between the South Asian subgroups, who traditionally have different dietary practices, Hindus being mostly vegetarian and Muslims being omnivorous (non-pork) eaters, while Punjabi Sikhs have few dietary restrictions. Only five Muslim participants attended the baseline studies during Ramadan fasting. Although adjustment for self-reported dietary intake did not affect the associations between AAs and incident diabetes, studies of the associations between dietary intake and AA levels have shown large diurnal and circadian variations and highly variable individual responses to different dietary components [38–42]. Further research is needed to understand the role of diet in the relationship between serum AA levels and risk of diabetes.

### Strengths and limitations

This is the first population-based study on AA profiles with a large number of South Asian migrants and native Europeans conducted in the same setting and with a lengthy follow-up. However, the study was conducted only in middle-aged men and the findings may not apply to women or other age groups.

Loss to follow-up occurred in approximately one-third of the participants, and while the baseline characteristics of the whole study group and those with follow-up data only were almost identical, it is likely that those lost to follow-up would be more at risk of developing diabetes and have more adverse outcomes, leading to a possible underestimation of the associations between AAs and incident diabetes. However, loss to follow-up did not differ by ethnicity, and it is unlikely that the strengths of the associations within ethnic groups would differ in those lost to follow-up.

We did not have baseline measures of hepatic function; hence we could not explore hepatic effects on AA levels and associations.

In addition, many associations have been compared within and between ethnic groups, and although the trends are consistent with previously published results in European populations, caution is needed in interpreting multiple comparisons.

Finally, comparisons of AA associations with incident diabetes within and between South Asian subgroups are based on small numbers and should be interpreted with caution.

### Summary and conclusions

Higher levels of isoleucine, phenylalanine, tyrosine, alanine and glutamine were observed in South Asian men. The associations between AAs and measures of obesity were generally less marked in the South Asian men. Levels of branched chain and aromatic AAs, particularly tyrosine, were adversely associated with incident diabetes to a greater extent in South Asian than in European men, even after adjustment for previously established risk factors, including insulin resistance and obesity. These findings suggest that higher branched chain and aromatic AAs, particularly tyrosine, may be a focus for identifying novel aetiological mechanisms and potential treatment targets for diabetes in South Asian individuals and may contribute to their excess risk of diabetes.

## Electronic supplementary material

Below is the link to the electronic supplementary material.

ESM Fig. 1 (PDF 85 kb)

ESM Table 1 (PDF 135 kb)

ESM Table 2 (PDF 150 kb)

## Abbreviations

AA: Amino acid
IDI: Integrated improvement index
Matsuda-IR: Matsuda index of insulin resistance
NMR: Nuclear magnetic resonance
NRI: Net reclassification index
SABRE: Southall And Brent REvisited
SHR: Sub-hazard ratio
