# Supplementary material for: Diabetes risk and amino acid profiles: cross-sectional and prospective analyses of ethnicity, amino acids and diabetes in a South Asian and European cohort from the SABRE (Southall And Brent REvisited) Study
Source: Diabetologia. 2015 Feb 19;58(5):968–79. doi: 10.1007/s00125-015-3517-8 (PMC4392114; doi:10.1007/s00125-015-3517-8)
Supplement: Supplementary file 1 — (PDF 85 kb) [file 125_2015_3517_MOESM1_ESM.pdf]

ESM Fig 1:

Follow-up of SABRE cohort (1988-2011). Incident diabetes and metabolomic analyses

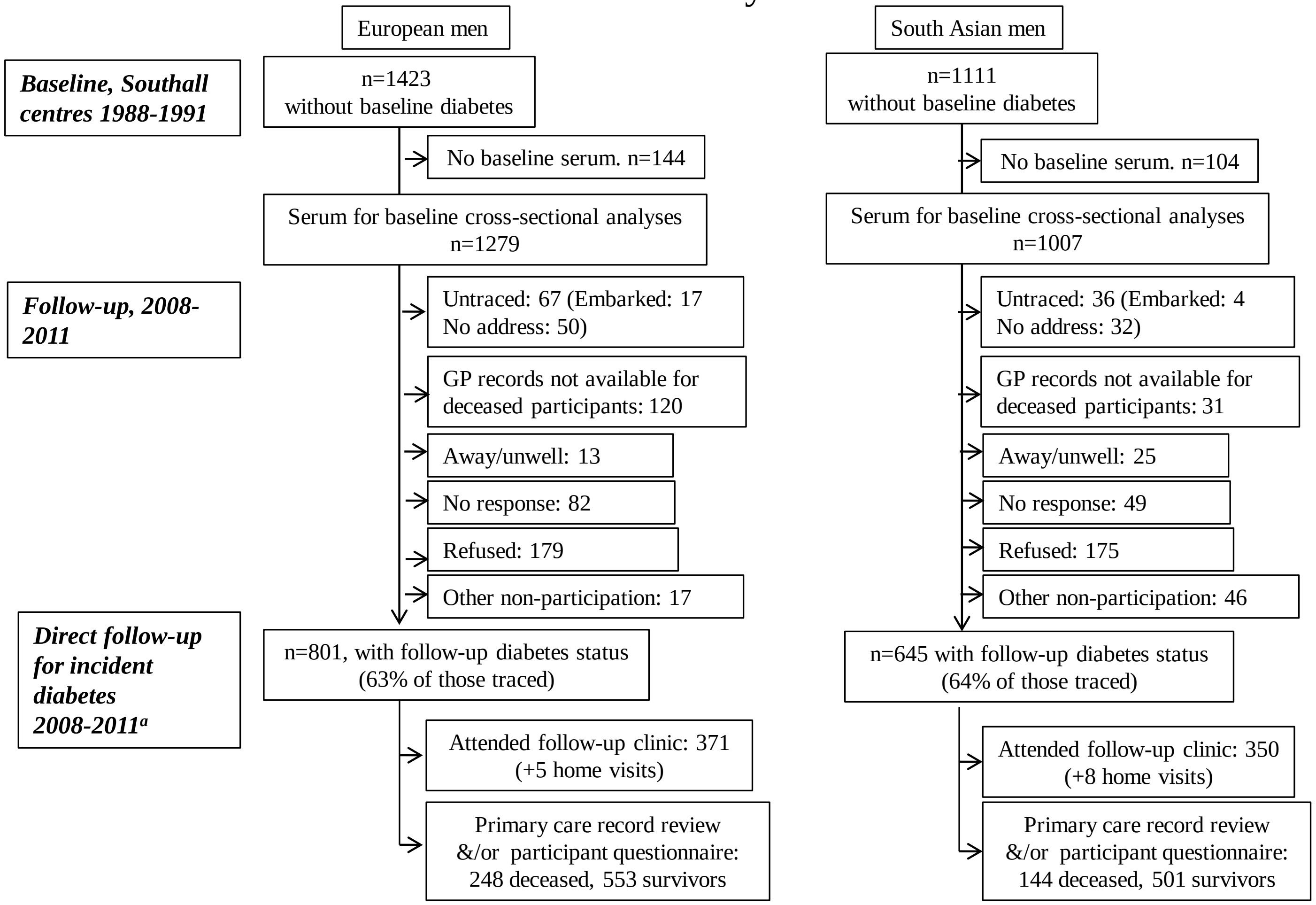

[a] Direct follow-up included clinic attendance/nurse home visit &/or primary care record review &/or participant questionnaire
