# Supplementary material for: Diabetes risk and amino acid profiles: cross-sectional and prospective analyses of ethnicity, amino acids and diabetes in a South Asian and European cohort from the SABRE (Southall And Brent REvisited) Study
Source: Diabetologia. 2015 Feb 19;58(5):968–79. doi: 10.1007/s00125-015-3517-8 (PMC4392114; doi:10.1007/s00125-015-3517-8)
Supplement: Supplementary file 2 — (PDF 135 kb) [file 125_2015_3517_MOESM2_ESM.pdf]

ESM Table 1. Associations between incident diabetes and amino acids (by quartiles). Logistic regression

Odd ratios	Quartile	Isoleucine	Leucine	Valine	Phenylalanine	Tyrosine	Isoleucine, phenylalanine and tyrosine	5 amino acids(isoleucine, leucine, valine, tyrosine, phenylalanine)
	Lowest quartile (reference)	1.00	1.00	1.00	1.00	1.00	1.00	1.00
Model 1	2nd quartile	1.65(0.86, 3.17)	1.64(0.83, 3.23)	1.26(0.65, 2.46)	0.63(0.34, 1.19)	1.50(0.82, 2.73)	1.64(0.88, 3.06)	1.95(1.03, 3.68)
European	3rd quartile	2.19(1.16, 4.14)	2.37(1.24, 4.53)	1.66(0.88, 3.12)	1.53(0.88, 2.66)	2.39(1.34, 4.28)	2.04(1.10, 3.79)	1.81(0.94, 3.48)
	4th quartile	3.48(1.90, 6.36)	3.34(1.82, 6.13)	2.46(1.35, 4.49)	1.43(0.83, 2.45)	3.07(1.71, 5.50)	3.56(2.00, 6.33)	3.64(2.00, 6.63)
Model 1	2nd quartile	1.20(0.71, 2.05)	1.25(0.75, 2.08)	1.18(0.73, 1.91)	2.52(1.53, 4.14)	1.33(0.70, 2.52)	1.50(0.82, 2.76)	1.49(0.85, 2.60)
South Asian	3rd quartile	2.22(1.35, 3.64)	2.47(1.51, 4.03)	1.89(1.19, 3.01)	1.48(0.90, 2.43)	3.03(1.68, 5.48)	3.57(2.03, 6.27)	3.66(2.18, 6.17)
	4th quartile	2.74(1.67, 4.48)	2.41(1.46, 3.99)	2.55(1.59, 4.07)	2.53(1.55, 4.15)	4.67(2.62, 8.28)	4.44(2.53, 7.81)	3.57(2.12, 6.02)
Model 2	2nd quartile	1.39(0.71, 2.69)	1.36(0.68, 2.70)	0.99(0.50, 1.98)	0.53(0.28, 1.02)	1.25(0.68, 2.30)	1.31(0.69, 2.48)	1.52(0.79, 2.91)
European	3rd quartile	1.70(0.89, 3.27)	1.78(0.92, 3.46)	1.11(0.57, 2.15)	1.15(0.65, 2.04)	1.67(0.91, 3.07)	1.39(0.73, 2.66)	1.26(0.64, 2.49)
	4th quartile	2.30(1.23, 4.33)	2.42(1.30, 4.52)	1.63(0.87, 3.06)	1.02(0.58, 1.80)	2.00(1.08, 3.69)	2.26(1.22, 4.17)	2.36(1.26, 4.42)
Model 2	2nd quartile	0.99(0.57, 1.71)	1.24(0.73, 2.09)	1.10(0.67, 1.80)	2.30(1.37, 3.86)	1.24(0.65, 2.39)	1.18(0.64, 2.21)	1.23(0.69, 2.17)
South Asian	3rd quartile	1.65(0.99, 2.76)	2.09(1.26, 3.48)	1.52(0.94, 2.47)	1.17(0.70, 1.96)	2.49(1.36, 4.57)	2.47(1.37, 4.44)	2.57(1.49, 4.42)
	4th quartile	1.78(1.06, 3.00)	1.82(1.07, 3.09)	1.94(1.18, 3.18)	1.79(1.06, 3.01)	3.25(1.78, 5.94)	2.79(1.54, 5.07)	2.39(1.38, 4.16)
Model 3	2nd quartile	1.10(0.55, 2.20)	1.24(0.61, 2.52)	1.03(0.51, 2.10)	0.48(0.25, 0.95)	1.09(0.58, 2.06)	1.01(0.52, 1.97)	1.20(0.61, 2.36)
European	3rd quartile	1.31(0.66, 2.58)	1.40(0.71, 2.78)	0.84(0.42, 1.71)	0.96(0.53, 1.76)	1.46(0.78, 2.73)	1.21(0.62, 2.35)	1.06(0.53, 2.12)
	4th quartile	1.65(0.85, 3.21)	1.90(0.99, 3.63)	1.34(0.69, 2.61)	0.78(0.43, 1.43)	1.43(0.74, 2.77)	1.50(0.78, 2.88)	1.62(0.83, 3.14)
Model 3	2nd quartile	0.88(0.50, 1.560	1.16(0.67, 2.01)	1.01(0.61, 1.69)	2.24(1.30, 3.86)	1.12(0.57, 2.21)	1.11(0.58, 2.10)	1.19(0.66, 2.16)
South Asian	3rd quartile	1.21(0.70, 2.10)	1.74(1.01, 2.97)	1.17(0.69, 1.96)	1.10(0.93, 0.99)	2.29(1.21, 4.31)	2.14(1.15, 3.96)	2.08(1.17, 3.70)
	4th quartile	1.34(0.77, 2.33)	1.73(0.99, 3.02)	1.64(0.97, 2.77)	1.60(0.92, 2.76)	2.79(1.49, 5.23)	2.15(1.14, 4.03)	1.96(1.09, 3.52)
Model 4	2nd quartile	0.87(0.49, 1.56)	1.34(0.65, 2.73)	1.11(0.54, 2.30)	0.45(0.23, 0.90)	1.06(0.55, 2.04)	0.92(0.46, 1.81)	1.21(0.61, 2.39)
European	3rd quartile	1.28(0.73, 2.24)	1.34(0.66, 2.70)	0.91(0.44, 1.87)	0.90(0.49, 1.67)	1.48(0.79, 2.79)	1.17(0.60, 2.31)	1.01(0.50, 2.06)
	4th quartile	1.23(0.70, 2.17)	2.01(1.04, 3.89)	1.40(0.71, 2.78)	0.77(0.42, 1.42)	1.56(0.80, 3.04)	1.52(0.79, 2.94)	1.67(0.85, 3.29)
Model 4	2nd quartile	1.08(0.54, 2.18)	1.23(0.70, 2.17)	0.99(0.59, 1.69)	1.98(1.13, 3.47)	1.14(0.56, 2.29)	1.13(0.58, 2.17)	1.25(0.68, 2.29)
South Asian	3rd quartile	1.28(0.64, 2.56)	1.69(0.98, 2.93)	1.28(0.75, 2.18)	0.93(0.53, 1.64)	2.22(1.15, 4.27)	2.06(1.09, 3.88)	1.97(1.09, 3.55)
	4th quartile	1.70(0.86, 3.32)	2.01(1.04, 3.89)	1.65(0.97, 2.82)	1.39(0.79, 2.45)	2.63(1.37, 5.03)	1.94(1.01, 3.71)	1.83(1.00, 3.34)
Model 5	2nd quartile	1.40(0.71, 2.75)	1.38(0.69, 2.78)	0.98(0.49, 1.96)	0.57(0.29, 1.09)	1.15(0.61, 2.14)	1.39(0.73, 2.66)	1.51(0.78, 2.93)
European	3rd quartile	1.80(0.93, 3.47)	1.78(0.91, 3.48)	1.09(0.56, 2.12)	1.12(0.62, 2.01)	1.58(0.86, 2.92)	1.41(0.74, 2.71)	1.27(0.64, 2.50)
	4th quartile	2.07(1.09, 3.94)	2.12(1.12, 4.01)	1.43(0.75, 2.70)	0.96(0.54, 1.71)	1.50(0.80, 2.83)	1.99(1.07, 3.67)	1.98(1.05, 3.76)
Model 5	2nd quartile	1.07(0.61, 1.88)	1.28(0.74, 2.19)	1.24(0.75, 2.06)	2.52(1.48, 4.29)	1.27(0.65, 2.47)	1.48(0.79, 2.79)	1.45(0.81, 2.60)
South Asian	3rd quartile	1.79(1.06, 3.03)	2.24(1.32, 3.80)	1.59(0.97, 2.61)	1.41(0.83, 2.39)	2.47(1.33, 4.57)	2.96(1.62, 5.37)	2.99(1.72, 5.20)
	4th quartile	2.09(1.23, 3.56)	2.06(1.20, 3.54)	1.98(1.20, 3.29)	2.19(1.29, 3.72)	3.08(1.67, 5.66)	3.11(1.71, 5.67)	2.55(1.46, 4.48)

Model 1: adjusted for age, Model 2: adjusted for age, waist:hip ratio, truncal skinfold thickness, Model 3: adjusted for age, waist:hip ratio, truncal skinfold thickness, Matsuda IR, HDL cholesterol, current smoking, Model 4: Model 3 plus alcohol consumption, Model 5: adjusted for age, fasting glucose, BMI (for comparison with the Framingham Offspring Study)

ESM Table 1(continued). Associations between incident diabetes and amino acids (by quartiles)

Odds ratios	Quartile	Alanine	Glutamine	Glycine	Histidine
	Lowest quartile (ref)	1.00	1.00	1.00	1.00
Model 1	2nd quartile	1.41(0.77, 2.58)	0.92(0.54, 1.56)	0.83(0.48, 1.42)	0.80(0.44, 1.44)
European	3rd quartile	1.48(0.82, 2.67)	0.82(0.48, 1.43)	0.79(0.46, 1.35)	1.30(0.76, 2.22)
	4th quartile	2.23(1.27, 3.91)	0.90(0.51, 1.61)	0.42(0.23, 0.78)	0.96(0.54, 1.70)
Model 1	2nd quartile	1.68(1.00, 2.83)	0.62(0.38, 1.00)	0.99(0.63, 1.55)	0.96(0.59, 1.56)
South Asian	3rd quartile	1.97(1.16, 3.33)	0.80(0.50, 1.29)	0.79(0.49, 1.25)	1.25(0.78, 2.02)
	4th quartile	3.01(1.81, 5.01)	0.67(0.43, 1.07)	0.96(0.61, 1.52)	1.33(0.83, 2.13)
Model 2	2nd quartile	1.22(0.66, 2.25)	0.86(0.50, 1.48)	0.87(0.50, 1.51)	0.83(0.45, 1.51)
European	3rd quartile	1.25(0.68, 2.28)	0.85(0.48, 1.48)	0.82(0.47, 1.42)	1.30(0.75, 2.25)
	4th quartile	1.6690.93, 2.97)	1.08(0.60, 1.97)	0.46(0.25, 0.86)	0.86(0.94, 1.00)
Model 2	2nd quartile	1.49(0.90, 2.56)	0.59(0.36, 0.99)	1.07(0.68, 1.71)	0.91(0.55, 1.51)
South Asian	3rd quartile	1.60(0.93, 2.76)	0.86(0.52, 1.41)	0.81(0.50, 1.32)	1.17(0.71, 1.92)
	4th quartile	2.32(1.37, 3.94)	0.82(0.51, 1.33)	1.05(0.65, 1.70)	1.20(0.73, 1.97)
Model 3	2nd quartile	1.15(0.61, 2.19)	0.95(0.54, 1.69)	0.78(0.43, 1.40)	0.84(0.45, 1.57)
European	3rd quartile	1.13(0.60, 2.12)	0.84(0.46, 1.52)	0.75(0.42, 1.33)	1.32(0.74, 2.36)
	4th quartile	1.39(0.75, 2.59)	1.11(0.59, 2.09)	0.44(0.22, 0.84)	0.84(0.45, 1.57)
Model 3	2nd quartile	1.50(0.86, 2.64)	0.56(0.33, 0.96)	1.16(0.71, 1.89)	1.02(0.60, 1.73)
South Asian	3rd quartile	1.39(0.78, 2.46)	0.90(0.53, 1.53)	0.84(0.51, 1.39)	1.23(0.73, 2.08)
	4th quartile	2.06(1.18, 3.59)	0.78(0.47, 1.31)	1.12(0.67, 1.86)	1.34(0.79, 2.25)
Model 4	2nd quartile	1.30(0.67, 2.52)	0.98(0.55, 1.76)	0.72(0.40, 1.32)	0.97(0.57, 1.68)
European	3rd quartile	1.34(0.69, 2.58)	0.78(0.42, 1.44)	0.76(0.42, 1.36)	1.31(0.77, 2.24)
	4th quartile	1.72(0.90, 3.28)	1.07(0.56, 2.04)	0.44(0.22, 0.85)	1.20(0.70, 2.06)
Model 4	2nd quartile	1.40(0.79, 2.49)	0.60(0.34, 1.04)	1.04(0.62, 1.73)	0.84(0.45, 1.58)
South Asian	3rd quartile	1.27(0.71, 2.27)	1.06(0.61, 1.85)	0.81(0.48, 1.36)	1.29(0.71, 2.32)
	4th quartile	1.75(0.99, 3.09)	0.97(0.56, 1.65)	1.09(0.65, 1.85)	0.84(0.45, 1.57)
Model 5	2nd quartile	1.15(0.62, 2.18)	0.88(0.50, 1.54)	0.95(0.53, 1.67)	0.84(0.45, 1.57)
European	3rd quartile	1.00(0.53, 1.87)	0.87(0.49, 1.55)	0.87(0.49, 1.54)	1.38(0.78, 2.43)
	4th quartile	1.31(0.72, 2.39)	1.19(0.65, 2.20)	0.48(0.25, 0.90)	0.81(0.45, 1.48)
Model 4	2nd quartile	1.31(0.76, 2.29)	0.65(0.39, 1.10)	1.08(0.67, 1.76)	0.91(0.54, 1.54)
South Asian	3rd quartile	1.44(0.82, 2.51)	0.89(0.53, 1.49)	0.79(0.48, 1.30)	1.20(0.72, 2.00)
	4th quartile	1.94(1.13, 3.36)	0.77(0.47, 1.27)	1.09(0.67, 1.79)	1.16(0.95, 1.93)
