# Supplementary material for: Diabetes risk and amino acid profiles: cross-sectional and prospective analyses of ethnicity, amino acids and diabetes in a South Asian and European cohort from the SABRE (Southall And Brent REvisited) Study
Source: Diabetologia. 2015 Feb 19;58(5):968–79. doi: 10.1007/s00125-015-3517-8 (PMC4392114; doi:10.1007/s00125-015-3517-8)
Supplement: Supplementary file 3 — (PDF 150 kb) [file 125_2015_3517_MOESM3_ESM.pdf]

ESM Table 2

Harrell's C statistic, Net Reclassification Index (NRI) and Integrated Improvement Index (IDI) for amino acid combinations and conventional risk factors in the prediction of incident diabetes

	European men	South Asian men
Model 1: adjusted for age, waist:hip ratio, truncal skinfold thickness, Matsuda IR, HDL cholesterol, current smoking		
C statistic	0.732	0.738
Model 1 plus tyrosine		
C statistic	0.731, p=0.7[b]	0.752, p=0.092[b]
IDI	0.0008, p=0.4[b]	0.023, p<0.0001[b]
NRI[a]	0.028, p=0.2[b]	0.098, p=0.0014[b]
Model 1 plus isoleucine, phenylalanine, tyrosine		
C statistic	0.732, p=1.0[b]	0.745, p=0.5[b]
IDI	0.0001, p=0.6[b]	0.019, p=0.0007[b]
NRI[a]	0.011, p=0.7[b]	-0.005, p=0.9[b]
Model 1 plus isoleucine, leucine, valine, phenylalanine, tyrosine		
C statistic	0.731, p=0.8[b]	0.743, p=0.4[b]
IDI	0.0004, p=0.5[b]	0.023, p=0.0002[b]
NRI[a]	0.008, p=0.8[b]	-0.022, p=0.5[b]

[a]NRI: Cutpoints for diabetes risk:15%/30%

[b]p value for comparison with model 1
